# A distinct phase of cyclin B (Cdc13) nuclear export at mitotic entry in *Schizosaccharomyces pombe*

**DOI:** 10.1098/rsob.250199

**Published:** 2025-09-17

**Authors:** Samir G. Chethan, Jessie M. Rogers, Drisya Vijayakumari, Wendi Williams, Vojislav Gligorovski, Sahand Jamal Rahi, Silke Hauf

**Affiliations:** ^1^ Department of Biological Sciences, Virginia Tech, Blacksburg, VA, USA; ^2^ Fralin Life Sciences Institute, Virginia Tech, Blacksburg, VA, USA; ^3^ Institute of Physics, École polytechnique fédérale de Lausanne (EPFL), Lausanne, Switzerland; ^4^ Center for the Mathematics of Biosystems, Virginia Tech, Blacksburg, VA, USA

**Keywords:** cell cycle, cyclin B, mitotic entry, nucleocytoplasmic transport, *Schizosaccharomyces pombe*

## Abstract

In eukaryotes, cell division requires coordination between the nucleus and cytoplasm. Entry into cell division is driven by cyclin-dependent kinases (CDKs), which need a cyclin binding partner for their activity. In *Schizosaccharomyces pombe* (fission yeast), the B-type cyclin Cdc13 is essential and sufficient for cell cycle progression and is strongly enriched in the nucleus. Here, we show that a fraction of Cdc13 is exported from the nucleus to the cytoplasm just prior to mitosis. This export could be critical to propagate CDK activity throughout the cell. Mutating three Cdc13 nuclear localization signals (NLSs) led to precocious enrichment of Cdc13 in the cytoplasm but did not accelerate mitotic entry, indicating that the export is not sufficient to trigger entry into mitosis. The export coincides with spindle pole body integration into the nuclear envelope and may be required to coordinate nuclear and cytoplasmic signalling required for this integration. The onset and stop of Cdc13 nuclear export are remarkably abrupt, underscoring that *S. pombe* mitotic entry consists of several switch-like transitions over the course of minutes. Our findings add another instance to the various cyclin nuclear transport events known to occur at critical cell cycle transitions throughout eukaryotes.

**Figure:**
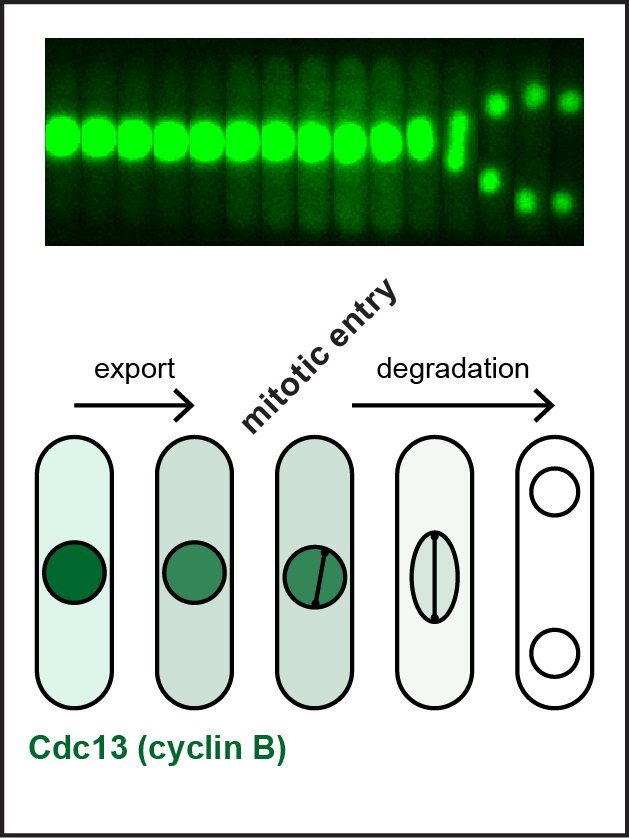

## 1. Background

The eukaryotic cell cycle is characterized by distinct phases (G1, S, G2 and M) with rapid transitions from G1 to S and G2 to M. The key drivers of the cell cycle are cyclin-dependent kinases (CDKs), which require binding to a cyclin protein for their activity [1–3]. In *Schizosaccharomyces pombe* (fission yeast), the cyclin-dependent kinase CDK1 (*S.p*. Cdc2) is essential for cell cycle progression and can pair with several cyclins [4]. The B-type cyclin Cdc13 is the only one of these cyclins that is essential for viability, and Cdc13 is sufficient to execute the cell cycle in the absence of the other Cdc2-binding cyclins [5,6]. Cdc13 accumulates during the S and G2 phases of the cell cycle and is ubiquitinated by the anaphase-promoting complex (APC/C) late in mitosis and G1, which targets it for proteasomal degradation. In all cell cycle phases where Cdc13 is present, it is strongly enriched in the nucleus [7–12].

The localization of cell cycle cyclins (and their CDK binding partners) is often dynamic [13]. *S.p*. Cdc13—in addition to its nuclear localization—binds to spindle pole bodies (SPBs) starting in late G2 phase and to the mitotic spindle during mitosis [12,14–19]. Cdc13 can also become enriched in the nucleolus at times [20–23]. In mammalian cells, cyclin A2 is mostly nuclear but becomes enriched in the cytoplasm after S phase and at centrosomes (equivalent to yeast spindle pole bodies) in early mitosis [24–31]. Mammalian cyclin B1 is mostly cytoplasmic but also localizes to centrosomes and is rapidly imported into the nucleus at entry into mitosis [24,25,32–34]. Cyclin B1’s nuclear import propagates CDK1 activity into the nucleus [34,35] and creates a spatial positive feedback loop, supporting a rapid and irreversible transition from G2 phase to mitosis [36,37]. In mitosis, cyclin B1 also localizes to spindle microtubules and kinetochores [24,32,36,38–41].

Here, we report that a fraction of *S. pombe* Cdc13 becomes exported from the nucleus just prior to mitosis, which has recently also been described by Kapadia & Nurse [42]. We find that Cdc13 export starts concomitant with Polo-like kinase (Plo1) enrichment at SPBs and stops when spindle pole bodies separate and the mitotic spindle forms. Along with other observations [42–48], our findings indicate that *S. pombe* mitotic entry is a fast sequence of at least three distinct switch-like events, probably coupled to the integration of the SPBs into the nuclear envelope.

## 2. Results

### 2.1. Cdc13 and Cdc2 export from the nucleus prior to degradation of Cdc13 in mitosis

**Figure 1.**
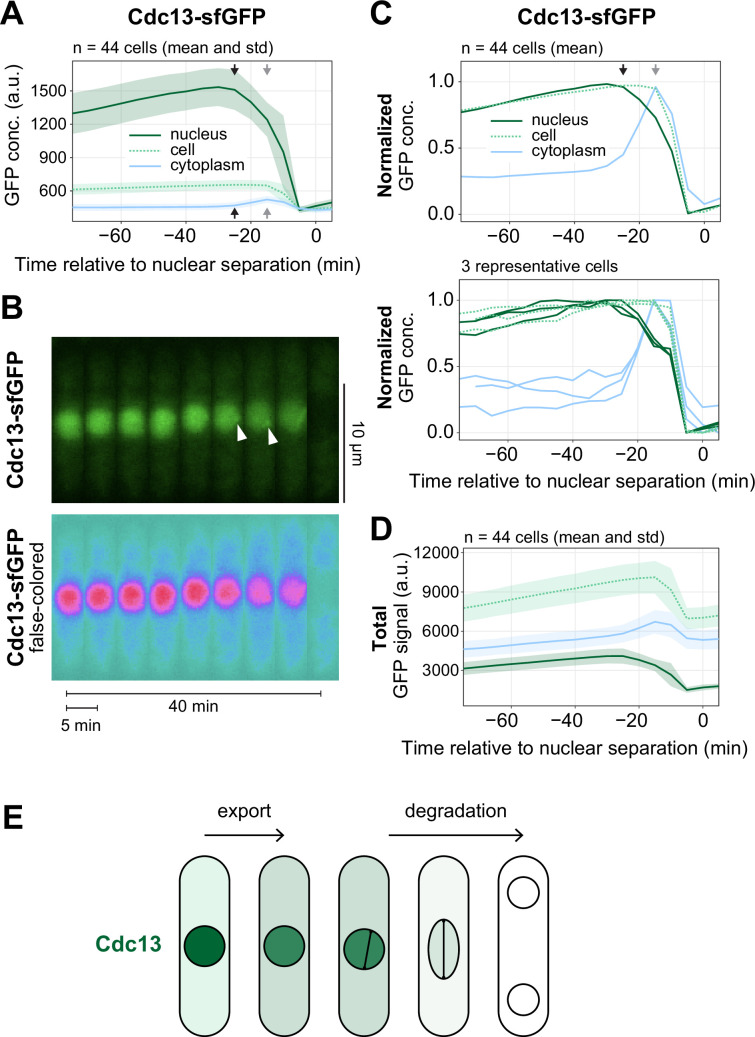
A fraction of Cdc13 exports from the nucleus prior to spindle formation. (A) Live-cell imaging of Cdc13-sfGFP, expressed from its endogenous genetic locus. Mean (line) and standard deviation (shaded area) of GFP concentrations in different compartments across 44 cells. Images were taken every 5 min. Curves were aligned to nuclear separation in anaphase (time 0). Cells were segmented based on the brightfield image, nuclei were segmented using an NLS-tdTomato signal. GFP concentrations are calculated as integrated signal per area. The cytoplasmic concentration was calculated from the difference in integrated signals between cell and nucleus divided by the area difference. Black arrows mark the approximate start of Cdc13 export from the nucleus, grey arrows mark the approximate start of Cdc13 degradation. (B) Kymograph of an exemplary cell from the experiment in (A). Arrowheads point to localized Cdc13 signals, probably colocalizing with SPBs. (C) GFP concentrations were normalized to the minimum and maximum signal within one cell cycle for each cell to better demonstrate the changes in whole cell and cytoplasmic GFP concentration. The mean across 44 cells (top) and three exemplary cells (bottom) are shown. (D) The integrated signal in different regions (mean and standard deviation) is shown rather than the concentration. (E) Schematic of Cdc13 nuclear export prior to spindle pole separation and Cdc13 degradation at the metaphase-to-anaphase transition.

When performing live-cell imaging of Cdc13-sfGFP, we observed a drop in nuclear concentration (figure 1A, black arrows) that occurred just prior to mitosis and distinctly prior to the degradation of Cdc13 in late mitosis (figure 1A, grey arrows). This drop is too subtle to be observed in population data when cells are arranged by size (electronic supplementary material, figure S1) [18] but has also been observed by others using live-cell imaging, either with the same or another Cdc13 tag [42,48]. The drop in nuclear concentration was initially interpreted as reflecting movement of Cdc13 to the SPB [48]. While SPB localization is visible in this period (figure 1B, arrowheads), we also detect a more general increase in cytoplasmic Cdc13 concentration in the same period, whereas the cellular concentration of Cdc13 stayed comparatively constant (figure 1A–C). Highly similar measurements were made by Kapadia & Nurse [42]. When we plotted the total amounts of Cdc13 in the cell, nucleus and cytoplasm, rather than the concentration, the cytoplasmic amount increased faster than the total amount and concomitant with a decrease in the nuclear amount (figure 1D). This indicates that a fraction of Cdc13 is exported from the nucleus at this time, prior to Cdc13 degradation in late mitosis (figure 1E). (Note that what we call ‘export’ could either be export or less efficient import of Cdc13 that cycles between nucleus and cytoplasm.)

**Figure 2.**
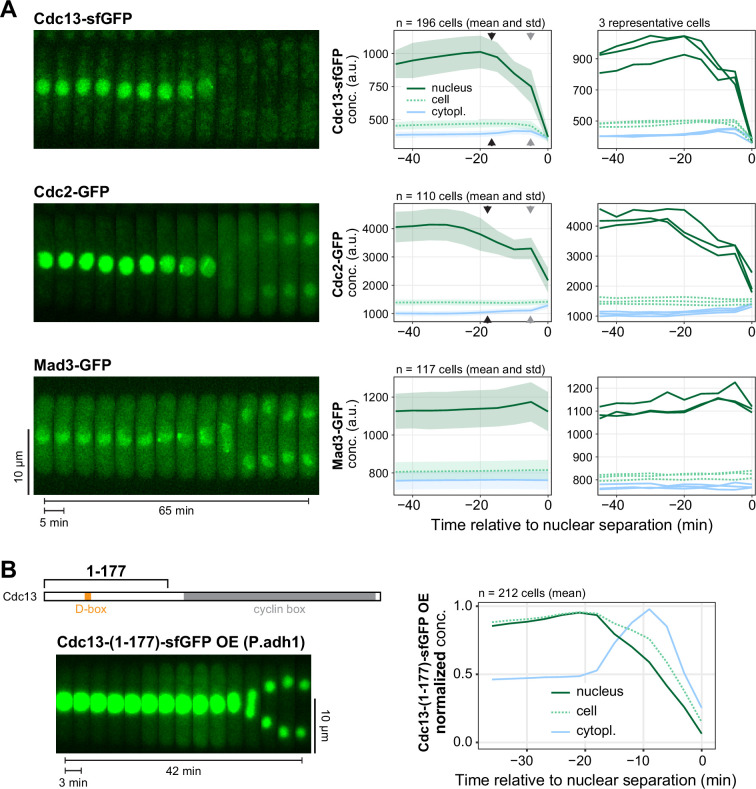
Cdc2 exports from the nucleus along with Cdc13, but Cdc13 can export without Cdc2. (A) Live-cell imaging of Cdc13-sfGFP, Cdc2-GFP and Mad3-GFP, similar to figure 1, all expressed from their endogenous genetic loci. Images were taken every 5 min. Kymographs from exemplary cells (left), mean and standard deviation of the GFP concentrations (middle) and GFP concentrations from three exemplary cells each (right). Black arrowheads mark the approximate start of Cdc13 and Cdc2 export from the nucleus, grey arrowheads mark the approximate start of Cdc13 degradation. Normalized GFP concentrations are shown in electronic supplementary material, figure S2. (B) Live-cell imaging of an N-terminal Cdc13 fragment (amino acids 1−177), C-terminally tagged with sfGFP and overexpressed (OE) from the strong *adh1* promoter at the *leu1* locus. Images were taken every 3 min. Imaging and analysis otherwise as in (A). Kymograph (left) and GFP concentration normalized to the minimum and maximum signal within one cell cycle (right).

Cdc13 binds Cdc2 and is required for the nuclear localization of Cdc2 [7,8,12]. We found that Cdc2 nuclear concentration also started to drop prior to mitosis, accompanied by an increase in cytoplasmic concentration (figure 2A, black arrowheads; electronic supplementary material, figure S2). An additional, further decrease in nuclear and increase in cytoplasmic Cdc2 concentration is observed later, when Cdc13 is degraded and no longer available as a binding partner (figure 2A, grey arrowheads; electronic supplementary material, figure S2). This suggests that Cdc13 and Cdc2 are exported from the nucleus as a complex, and therefore that CDK1 activity may spread to the cytoplasm during this time. As a control, another nuclear-enriched protein (Mad3, a spindle assembly checkpoint protein) did not show a drop in nuclear concentration during that same period (figure 2A; electronic supplementary material, figure S2), indicating that the export is actively regulated rather than representing unspecific leakiness of the nuclear envelope. Leakiness may have occurred because the *S. pombe* SPB integrates into the nuclear envelope during this time, which requires nuclear envelope fenestration [49,50].

Even though Cdc13 and Cdc2 may export as a complex, the export of Cdc13 does not require Cdc2, since the N-terminal unstructured region of Cdc13, Cdc13-(1-177), which is not expected to interact with Cdc2 [38,51,52], shows the same brief period of nuclear export (figure 2B). (Note that Cdc13-(1-177) is overexpressed in this experiment, making the increase in cytoplasmic concentration more obvious.)

### 2.2. Cdc13 export takes place prior to spindle pole body separation and is concomitant with Plo1 enrichment at spindle pole bodies

**Figure 3.**
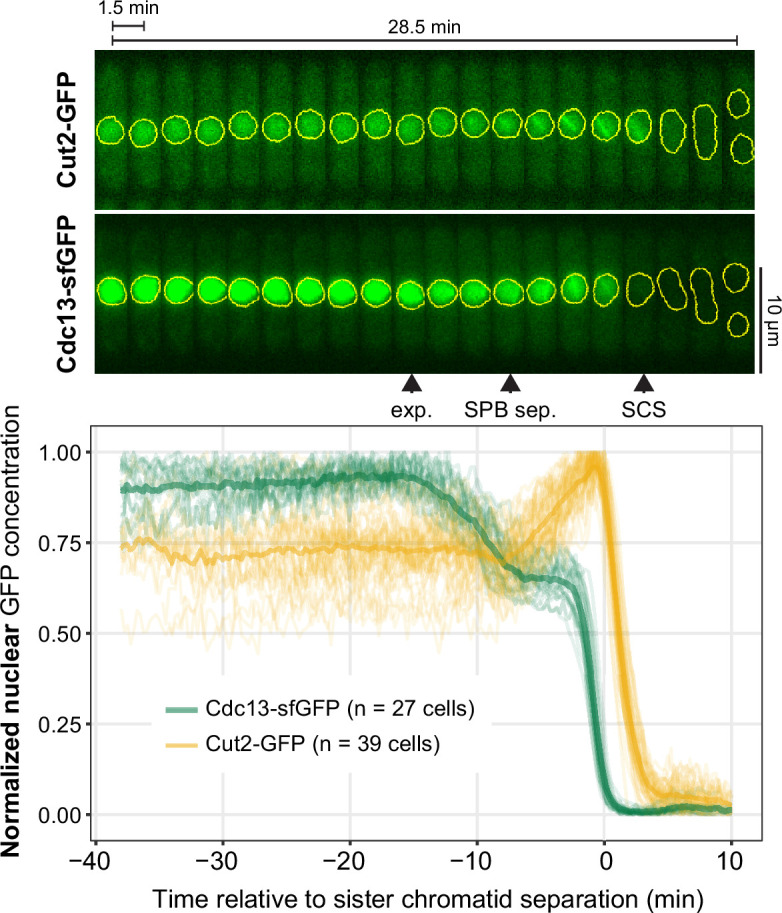
Cdc13 stops exporting from the nucleus at spindle formation. Live-cell imaging of Cdc13-sfGFP and Cut2-GFP, both expressed from their endogenous genetic loci. Images were taken every 15 s; the kymographs from exemplary cells (top) only show every sixth image, yellow shapes indicate the area segmented as nucleus. Arrowheads mark the approximate positions of start of Cdc13 nuclear export (exp.), SPB separation (SPB sep.) and sister chromatid separation (SCS). The centromeres of chromosome 1 were labelled with tdTomato (electronic supplementary material, figure S3), and nuclear GFP concentration curves were aligned to sister chromatid separation in anaphase (time 0). Nuclear concentrations (bottom) of individual cells (*n* = 27 for Cdc13 and *n* = 39 for Cut2) as thin lines, their mean as thick line. Concentrations were normalized to the maximum and minimum for each cell. To make the strains as similar as possible, Cut2 is tagged with non-fluorescent GFP in the Cdc13-sfGFP strain, and Cdc13 is tagged with non-fluorescent sfGFP in the Cut2-GFP strain. Exemplary single cells are shown in electronic supplementary material, figure S4.

To better assess the timing of Cdc13 export, we co-labelled the centromere of chromosome 1 with tdTomato, took images every 15 s and aligned the measurements to sister chromatid separation in anaphase (figure 3**,** electronic supplementary material, figure S3). Nuclear export of Cdc13 began about 15 min prior to anaphase and, remarkably, ceased about 7 min prior to anaphase, which corresponds to the time when spindle poles separate. Another APC/C substrate, securin (*S.p*. Cut2), showed a different pattern: while Cdc13 was exporting, Cut2 nuclear concentration stayed constant, but it imported into the nucleus starting at SPB separation (figure 3). This confirms that Cdc13 nuclear export is not a consequence of nuclear leakiness and indicates two different phases of nuclear transport regulation at entry into mitosis: before and after spindle pole separation.

At this higher temporal resolution, it became apparent that the onset and stop of nuclear export are rapid events, observable by the abrupt slope change both in the averaged data (figure 3) as well as in single cells (electronic supplementary material, figure S4). The transition to decreasing nuclear Cdc13 concentration happens within about a minute, and the transition from export to its cessation is typically even more abrupt (electronic supplementary material, figure S4). This suggests that the onset and stop of nuclear export are switch-like transitions and require a type of molecular regulation that can implement such rapid change.

**Figure 4.**
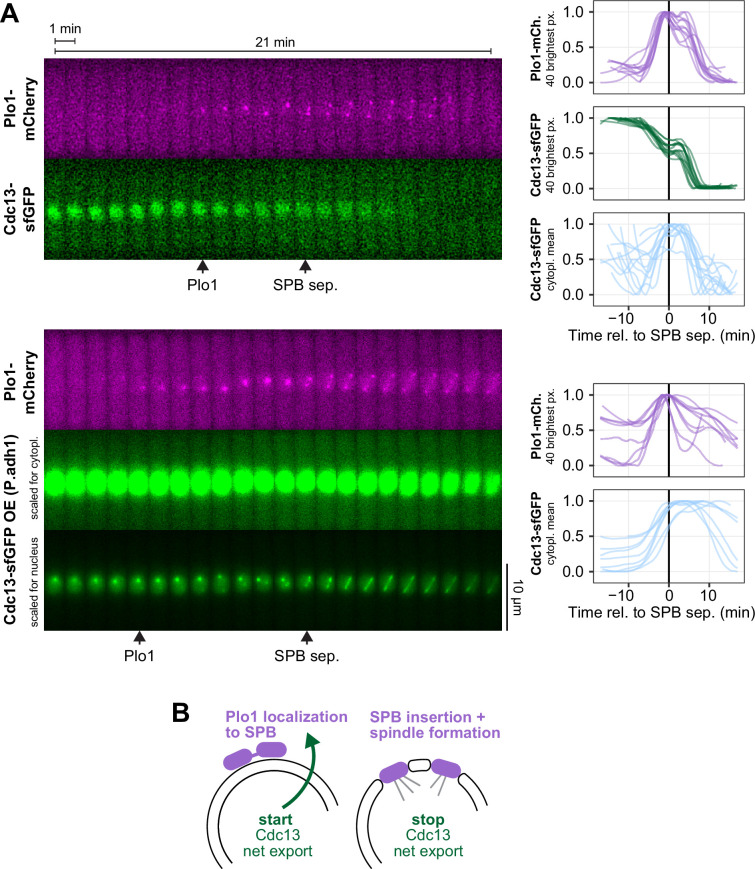
Cdc13 nuclear export is concomitant with Plo1 enrichment at the spindle pole bodies. (A) Live-cell imaging of Plo1-mCherry and Cdc13-sfGFP, both expressed from their endogenous genetic locus. Cdc13-sfGFP expressed from the *cdc13* promoter (top) or the strong *adh1* promoter (bottom). Images were taken every 15 s; the kymographs from exemplary cells (left) only show every fourth image. Graphs on the right show concentrations from individual cells normalized to the minimum and maximum signal recorded for each cell (*n* = 12 and *n* = 9 cells, respectively). Curves were aligned to SPB separation (time 0). Since the strains did not express a nuclear marker, the concentration of Cdc13 in the nucleus was estimated from the mean of the 40 brightest pixels in the cell. This was not possible for the overexpression strain due to strong accumulation of the overexpressed Cdc13 at a dot-like spot within the nucleus, probably the rDNA (bottom panel in bottom kymograph). This signal was so strong that it also bled through into the mCherry channel (seen as very weak dot-like signal in the images just before Plo1-mCherry starts to concentrate at the spindle pole bodies). The mean of the 40 brightest pixels was also used to quantify the accumulation of Plo1-mCherry at the spindle pole bodies. GFP concentration in the cytoplasm (cytopl. mean) was measured from two manually placed regions on either side of the nucleus. Arrowheads on the kymographs mark the approximate positions of the start of Plo1 enrichment at the SPB region (Plo1) and SPB separation (SPB sep.). Cdc13 overexpression (approx. 20×, bottom panel) can cause delays in mitosis, leading to the persistence of Cdc13-sfGFP beyond 10 min seen for some cells quantified in the bottom panel. (B) Schematic illustrating that nuclear export of Cdc13 starts concomitantly with the enrichment of Plo1 at SPBs and stops concomitantly with SPB separation.

To position the onset of Cdc13 nuclear export relative to other events, we looked at Plo1 localization. Plo1 (Polo or Plk1 in other organisms) is a key kinase in the regulation of mitotic entry in both mammalian cells and *S. pombe* [53–57]. In *S. pombe*, Plo1 becomes enriched at SPBs in late G2, just prior to mitosis, and SPBs serve as a signalling hub for mitotic entry regulation involving Plo1 and Cdc2 [12,17,19,57–61]. We imaged Plo1-mCherry together with Cdc13-sfGFP. In these experiments, Cdc13-sfGFP was expressed from the endogenous locus either under the endogenous *cdc13* promoter or overexpressed under the *adh1* promoter (figure 4A). These experiments confirmed that Cdc13 nuclear export takes place prior to SPB separation and showed that its start is concomitant with the enrichment of Plo1 at SPBs and stops with SPB separation (figure 4A,B). Fission yeast SPBs are located outside the nuclear envelope during interphase and need to integrate into the nuclear envelope for mitotic spindle formation [49,50,62,63]. The coincidence in timing suggests that Cdc13 export is important for SPB integration.

### 2.3. Movement of Cdc13 from nucleus to cytoplasm is not sufficient for mitotic entry

**Figure 5.**
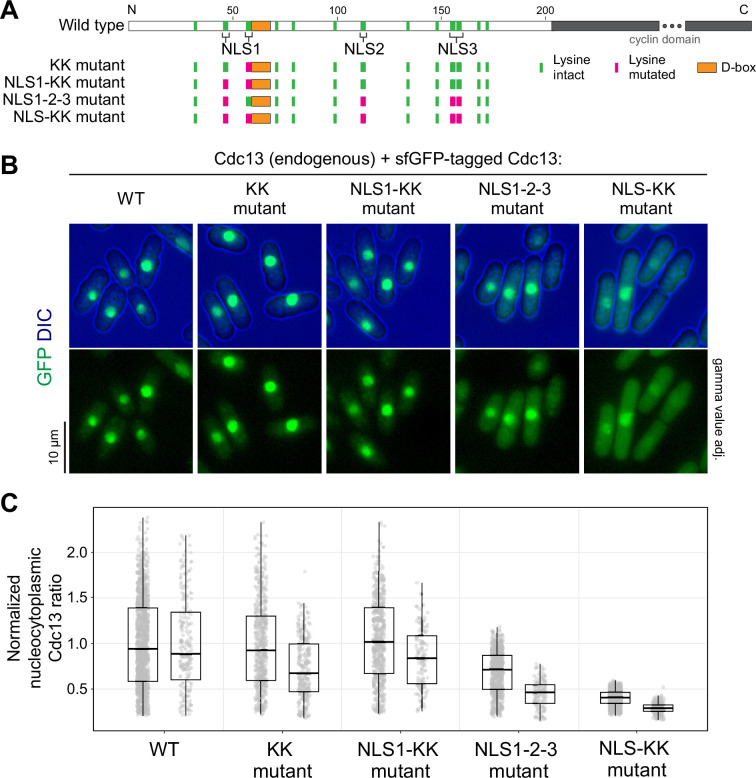
Mutation of three Cdc13 nuclear localization signals causes accumulation of Cdc13 in the cytoplasm. (A) Schematic depicting the location of lysine residues and the D-box within the Cdc13 protein disordered region. Residues mutated in respective constructs are highlighted in magenta. (B) Representative images of asynchronous cultures of cells expressing wild-type or mutant versions of Cdc13-sfGFP expressed from the exogenous *leu1* locus under the endogenous *cdc13* promoter. In images showing GFP only, the gamma values were adjusted to better illustrate the range of signals. (C) Quantification of the nucleocytoplasmic ratio of Cdc13 concentration for wild-type Cdc13 and NLS mutant versions. Two independent experiments, imaged on different microscopes. Nucleocytoplasmic Cdc13 ratios for each cell were normalized to the mean nucleocytoplasmic ratio of Cdc13 in wild-type cells within each respective experiment. *n* > 550 and >120 cells per strain in experiments 1 and 2, respectively. Individual cells (circles) and summary data; box: median and quartiles; whiskers: 1.5 times interquartile range.

**Figure 6.**
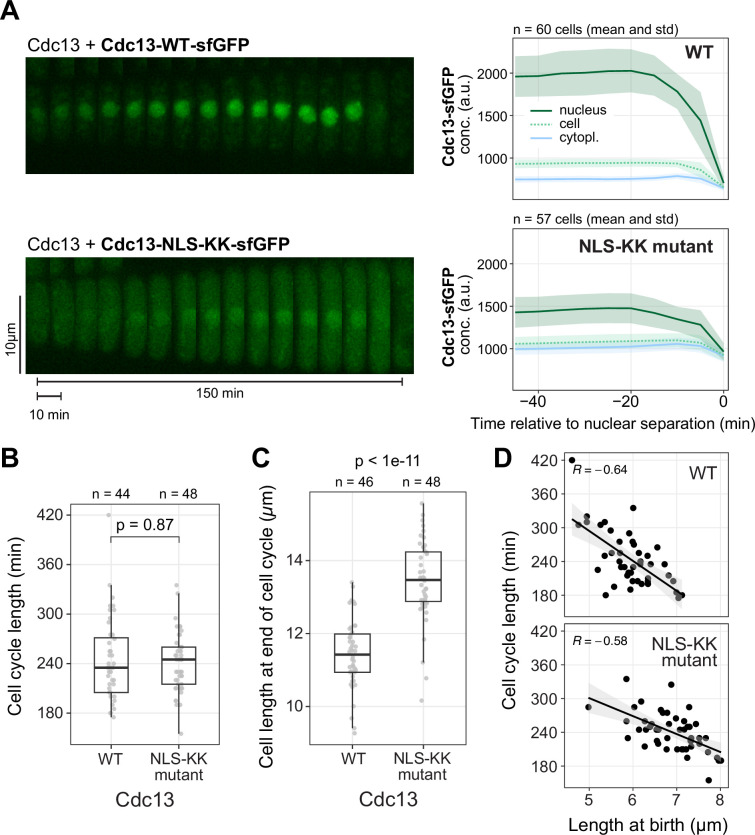
Cdc13 nuclear export is not the rate-limiting step for entry into mitosis. (A) Live-cell imaging of wild-type (WT) or NLS-KK mutant Cdc13, similar to figure 1, except that the fluorescently tagged versions were expressed from the exogenous *leu1* locus under the *cdc13* promoter and untagged, wild-type *cdc13* is expressed from its endogenous locus. Images were taken every 5 min. Kymographs from exemplary cells (left), mean and standard deviation of the GFP concentrations (right). Plots for the normalized and total GFP signal are available in electronic supplementary material, figure S6. (B,C) Time from one cell division to the next (B) and cell length in the time frame prior to cell division (C) from the live-cell imaging experiment in (A). Individual cells (circles) and summary data; box: median and quartiles; whiskers: 1.5 times interquartile range. *p*-values from Wilcoxon rank sum test with continuity correction. (D) Cell length at birth versus length of the subsequent cell cycle from the live-cell imaging experiment in (A). Individual cells (circles) and linear regression. *R*, Pearson correlation coefficient.

To address whether the export of Cdc13 from the nucleus influences the timing of entry into mitosis, we sought to precociously enrich Cdc13 in the cytoplasm. The N-terminal unstructured region of Cdc13 contains three candidate nuclear localization signals (NLSs) (figure 5A, electronic supplementary material, figure S5) [8]. We mutated these and expressed the mutant constructs under the endogenous regulatory sequences from an exogenous locus, leaving the endogenous *cdc13* intact. While mutation of individual NLSs did not prominently affect Cdc13 nucleocytoplasmic distribution (figure 5B,C, and not shown), combining mutations in all three NLSs led to a reduction in nuclear enrichment (figure 5B,C, NLS1-2-3 mutant). We initially left two lysine residues that are part of the predicted bi-partite NLS1 intact because they are directly adjacent to the destruction box (D-box), and we assumed they may be important targets for ubiquitination and therefore essential for the rapid proteasome-mediated degradation of Cdc13 in late mitosis. However, mutation of these residues still allowed for degradation of Cdc13 at the end of mitosis while further impairing nuclear enrichment (figures 5B,C and 6A, electronic supplementary material, figure S6A,B, NLS-KK mutant). In live-cell imaging, a prominent cytoplasmic pool of Cdc13-NLS-KK was observed throughout the cell cycle (figure 6A**,** electronic supplementary material, figure S6B). Despite the precocious and strong enrichment of Cdc13-NLS-KK in the cytoplasm, the length of the cell cycle was not shortened in this strain (figure 6B), indicating that Cdc13 nuclear export is not sufficient to trigger mitotic entry. This situation is similar to that of mammalian cyclin B1, which rapidly imports into the nucleus at entry into mitosis, but in cell lines, a constitutive or precocious nuclear localization of cyclin B1 does not accelerate entry into mitosis [64–67].

**Figure 7.**
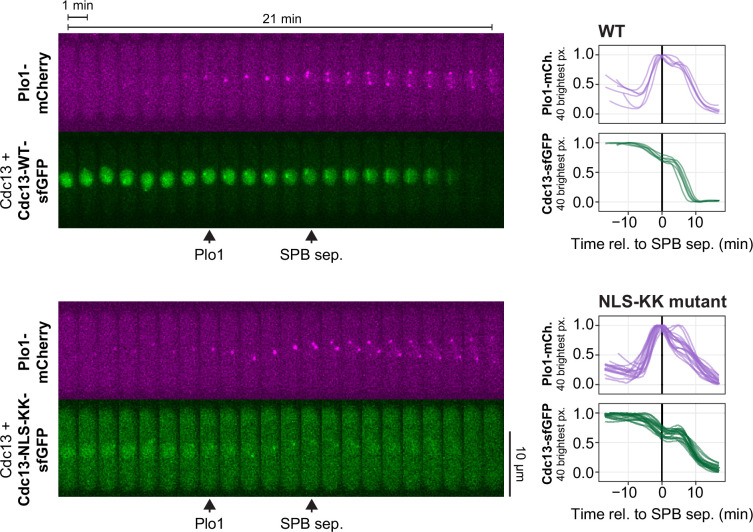
Plo1 enrichment at spindle pole bodies is not accelerated by cytoplasmic Cdc13 in the NLS-KK mutant. Live-cell imaging of Plo1-mCherry, expressed from its endogenous locus, and sfGFP-tagged wild-type Cdc13 or NLS-KK mutant, expressed from the exogenous *leu1* locus under the *cdc13* promoter. Untagged, wild-type *cdc13* is expressed from its endogenous locus. Images were taken every 15 s; the kymographs from exemplary cells (left) show every fourth image. Arrowheads on the kymographs mark the approximate positions of the start of Plo1 enrichment at the SPB region (Plo1) and SPB separation (SPB sep.). Graphs on the right show concentrations from individual cells normalized to the minimum and maximum signal recorded for the cell (*n* = 6 and *n* = 16 cells from two independent strains, respectively). Curves were aligned to SPB separation (time 0). The mean of the 40 brightest pixels in the cell was used to quantify the accumulation of Plo1-mCherry at the spindle pole bodies and obtain an estimate for the nuclear Cdc13 concentration. Plots for the non-normalized concentrations and additional kymographs are shown in electronic supplementary material, figure S7.

Consistent with the unaltered cell cycle length, we found that strong enrichment of Plo1 at SPBs remained restricted to approximately 8 min prior to SPB separation in cells expressing Cdc13-NLS-KK (figure 7**,** electronic supplementary material, figure S7). Plo1 enrichment still coincided with Cdc13 nuclear export. Nuclear export was detectable as a drop in nuclear signal in the Cdc13-NLS-KK mutant despite its lowered nucleocytoplasmic ratio (figure 7, electronic supplementary material, figure S7). This suggests that the change in nucleocytoplasmic Cdc13 distribution prior to mitosis is not a consequence of blocking NLS function—at least not of those NLSs that are mutated in Cdc13-NLS-KK. What mediates the remaining nuclear enrichment of Cdc13-NLS-KK is unknown. Cdc13 does not contain other classical NLSs. Nuclear accumulation of Cdc13 therefore may partially rely on non-canonical mechanisms such as direct binding to importin-β, as is the case for other cyclins [27,68–70].

Taken together, the unaltered cell cycle length and similar kinetics of Plo1 accumulation at SPBs in Cdc13-NLS-KK-expressing cells suggest that increasing CDK1 activity in the cytoplasm is insufficient to trigger mitotic entry.

While the cell cycle length of Cdc13-NLS-KK mutant-expressing cells was unchanged, the size of these cells increased (figure 6C; electronic supplementary material, figure S6C). Size homeostasis (longer cell cycles in cells that are born shorter [71]) was maintained (figure 6D; electronic supplementary material, figure S6D,E), indicating that the ‘set point’ for size had changed without altering the regulation of size control. We attribute this to the altered distribution of Cdc13 (and therefore probably Cdc2) between the nucleus and cytoplasm. With less nuclear CDK1 activity, cells may reach the threshold for mitotic entry only at a larger size. The alteration of cell size is consistent with observations that perturbing the nucleocytoplasmic ratio of the Cdc2 regulators Wee1 or Cdc25 alters cell size [45,72,73].

## 3. Discussion

**Figure 8.**
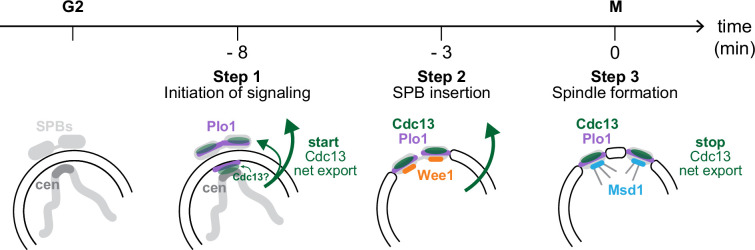
Cdc13 export is part of a stepwise entry into mitosis, coincident with SPB insertion into the nuclear envelope. Model for multiple steps at the *S. pombe* G2/M transition. The start of Cdc13 nuclear export coincides with Plo1 and Cdc13 enrichment at the SPB region. SPB integration into the nuclear envelope requires centromere (cen) contact with the SPB region [46,47]. Plo1 localizes both to cytoplasmic SPBs and to the nucleoplasmic side of the nuclear envelope adjacent to the SPBs [47]. The precise Cdc13 localization in this time window has not been determined. Cdc13 nuclear export could be required to bring active Cdc13/Cdc2 into contact with Plo1 at the cytoplasmic SPBs to jointly orchestrate SPB insertion into the nuclear envelope. Wee1 localizes to the SPBs distinctly later than Plo1 [45], possibly when SPBs have become integrated into the nuclear envelope and thus become accessible to Wee1, which is enriched in the nucleus. Cdc13 export stops around the time of spindle formation. Wee1 dissociates from SPBs at this time and other factors, such as Msd1, bind. Each transition step is fast, indicating that they involve ultrasensitivity in the underlying regulation.

The nucleus of eukaryotic cells provides additional opportunities for the regulation of cellular activities but also brings about the need to coordinate nuclear and cytoplasmic events. Changes in the nucleocytoplasmic distribution of cyclins or other CDK regulators are major themes in cell cycle regulation. Here, we show that a pool of *S. pombe* Cdc13 translocates from the nucleus to the cytoplasm at about the time when Plo1 starts to become strongly enriched at SPBs. We suggest that this is part of a sequence of events leading to the insertion of the SPBs into the nuclear envelope and, ultimately, mitotic spindle formation (figure 8). The export could serve to spread CDK1 activity into the cytoplasm, as also proposed by Kapadia & Nurse [42], who have shown, using biosensors for CDK1 activity, that CDK1 becomes active in the nucleus prior to Cdc13 export and prior to detectable CDK1 activity in the cytoplasm. The Cdc13 translocation seems functionally analogous to the movement of vertebrate cyclin B1 at entry into mitosis [34], except that the directionality is reversed (vertebrate cyclin B1 moving from the cytoplasm to the nucleus, and *S. pombe* Cdc13 moving in the other direction).

### 3.1. The switch-like onset and stop of Cdc13 nuclear export could be implemented by phosphoregulation

The mechanism by which the change in nucleocytoplasmic distribution of Cdc13 occurs is still unknown. One possibility is post-translational regulation, such as phosphorylation, which would be dynamic enough to change the nucleocytoplasmic distribution within minutes. The onset and stop of Cdc13 export are strikingly switch-like (figure 3; electronic supplementary material, figure S4), which suggests ultrasensitivity in the underlying regulation and could, for example, be implemented by multi-site phosphorylation or enzyme saturation [74,75]. Multiple phosphorylation sites have been identified in the N-terminal, unstructured region of Cdc13 that also contains the NLSs [22,76–81] (electronic supplementary material, figure S5A), but the role of most of these sites has not yet been tested. Mutation of three phosphorylated Cdc13 residues downstream of the NLSs (S177, S180 and S183) did not interfere with Cdc13 localization or function [22]. It is also possible that import or export regulators are post-translationally modified. For vertebrate cyclin B1’s rapid import into the nucleus at entry into mitosis, phosphorylation of both cyclin B1 and transport effectors has been implicated [33,34,64,82–84]. In *S. pombe*, the phosphorylation of several nucleoporins increases around M-phase, which could alter nuclear transport [79,85,86]. Furthermore, reducing the dosage of several nucleoporins has been shown to increase cell size in *S. pombe*, similar to *cdc13, cdc2* and *cdc25* mutants [87]. Whether this is related to altered Cdc13 nuclear export remains to be tested.

### 3.2. Cdc13 export may propagate CDK1 activity to the cytoplasm to coordinate stepwise SPB integration

SPBs and their vertebrate counterparts, centrosomes, are considered hubs of mitotic entry regulation [53,56,88]. In *S. pombe*, the SPBs are located adjacent to, but outside, the nuclear envelope during interphase and only become integrated into the nuclear envelope at the onset of mitosis [50,62,89]. Their proper integration is required for the formation of the mitotic spindle [43,63,90,91]. Plo1, localized to SPBs, is needed for the integration of the SPBs into the nuclear envelope [47,60,92,93], and Cdc2, in turn, is required for Plo1 localization and activity at SPBs [59,61,94,95]. We consider it plausible that Cdc2/Cdc13 export from the nucleus to the cytoplasm is required to initiate SPB integration into the nuclear envelope by co-positioning Plo1 and Cdc13/Cdc2 at the cytoplasmic SPBs (figure 8). Contact between centromeres and the nuclear envelope beneath the SPBs is also required for SPB insertion, and this signalling has also been suggested to involve Plo1 and possibly Cdc2 [46,47]. Hence, both nuclear and cytoplasmic Cdc2 activity could be important, and Cdc13 nuclear export may be required to provide them both. Interestingly, mutation of the hydrophobic patch of Cdc13 has been shown to prevent not only Cdc13 enrichment in the SPB region [17,19] but also Cdc13 nuclear export [42], which suggests positive feedback between localized CDK1 activity at the SPB region and Cdc13 export. The binding partner for the hydrophobic patch has not been identified, and it is unclear whether this reflects a defect in a nuclear or cytoplasmic interaction.

The start of Cdc13 export coincides with Plo1 enrichment at the SPB region and its stop with SPB separation (figure 4). Live-cell imaging of mitotic entry regulators by Masuda *et al*. [45] has uncovered one additional switch-like step between the enrichment of Plo1 at SPBs and SPB separation: the CDK1-inhibiting kinase Wee1 and the kinesin Cut7 become enriched at SPBs about 3–4 min prior to SPB separation [45], clearly after the start of Plo1 enrichment at about 6–8 min prior to SPB separation (figure 8). We suggest that the first wave of binding (Plo1 and Cdc13) corresponds to preparing the SPB for insertion, and the second wave (Wee1 and Cut7) corresponds to insertion into the nuclear envelope, which makes the SPB accessible to nuclear proteins such as Wee1. At SPB separation, Wee1 is removed from SPBs, and the microtubule-anchoring protein Msd1 binds [45]. This final step coincides with the cessation of Cdc13 export. Thus, *S. pombe* mitotic entry consists of at least three switch-like transitions, tightly linked to SPB events and bracketed by Cdc13 nuclear export. We propose that the export is required to provide both nuclear and cytoplasmic activities for SPB integration.

## 4. Material and methods

### 4.1. Strains and growth

**Table:** 

strain name	mating type	genotype	figure	short name
SW643	*h?*	*cdc13-S177S-sfGFPcp zfs1+::natR:P.adh31-tetR-tdTomato*	figures 1 and 2; electronic supplementary material, figure S1 and S2	Cdc13-sfGFP
SX387	*h+*	*cdc2-GFP:kanMX6 zfs1+::natR:P.adh31-tetR-tdTomato cdc13-S177S-(sfGFPcp-Y138L*)	figure 2; electronic supplementary material, figure S2	Cdc2-GFP
SW182	*h+*	*mad3-ymEGFP zfs1+::natR:P.adh31-tetR-tdTomato*	figure 2; electronic supplementary material, figure S2	Mad3-GFP
SU425’	*h-*	*ura4? leu1-32::P.adh1-cdc13(1-177)-sfGFPcp:leu1+ dh1L::ura4+:tetO zfs1+::natR:P.adh31-tetR-tdTomato*	figure 2	Cdc13- (1-177)-sfGFP OE (P.adh1)
ST961	*h-*	*leu1-32 cdc13-S177S-sfGFPcp cut2-(GFP-Y66L):kanR dh1L::ura4+:tetO zfs1+::natR:P.adh31-tetR-tdTomato*	figure 3; electronic supplementary material, figures S3 and S4	Cdc13-sfGFP
SW058	*h-*	*leu1-32 cdc13-S177S-(sfGFPcp-Y138L) cut2-GFP:kanR dh1L::ura4+:tetO zfs1+::natR:P.adh31-tetR-tdTomato*	figure 3; electronic supplementary material, figures S3 and S4	Cut2-GFP
ST966	*h-*	*cdc13-S177S-sfGFPcp plo1-mCherry:natR*	figure 4	Cdc13-sfGFP
ST967	*h+*	*leu1-32 cdc13-S177S-sfGFPcp plo1-mCherry:natR*	figure 4	Cdc13-sfGFP
SU417	*h+*	*natNT2:P.adh1-cdc13-S177S-sfGFPcp plo1-mCherry:natR*	figure 4	Cdc13-sfGFP OE (P.adh1)
SU418	*h-*	*leu1-32 natNT2:P.adh1-cdc13-S177S-sfGFPcp plo1-mCherry:natR*	figure 4	Cdc13-sfGFP OE (P.adh1)
SW662	*h-*	*leu1-32::P.cdc13-cdc13-S177S-sfGFPcp:leu1+ zfs1+::natR:P.adh31-tetR-tdTomato*	figures 5 and 6; electronic supplementary material, figure S6	WT
SX560	*h-*	*leu1-32::P.cdc13-cdc13-KK57AS-S177S-sfGFPcp:leu1+ zfs1+::natR:P.adh31-tetR-tdTomato*	figure 5	KK mutant
SX561	*h-*	*leu1-32::P.cdc13-cdc13-KK46AS-KK57AS-S177S-sfGFPcp:leu1+ zfs1+::natR:P.adh31-tetR-tdTomato*	figure 5	NLS1-KK mutant
SX534	*h-*	*leu1-32::P.cdc13-cdc13-KK46AS-KKRR112ASSA-KKLKK155ASLAA-S177S-sfGFPcp:leu1+ zfs1+::natR:P.adh31-tetR-tdTomato*	figure 5	NLS1-2-3 mutant
SX562	*h-*	*leu1-32::P.cdc13-cdc13-KK46AS-KK57AS-KKRR112ASSA-KKLKK155ASLAA-S177S-sfGFPcp:leu1+ zfs1+::natR:P.adh31-tetR-tdTomato*	figures 5 and 6; electronic supplementary material, figure S6	NLS-KK mutant
SX585	*h+*	*ade6-M210 leu1-32::P.cdc13-cdc13-S177S-sfGFPcp:leu1+ plo1-mCherry:natR*	figure 7; electronic supplementary material, figure S7	WT
SX586	*h+*	*ade6-M210 leu1-32::P.cdc13-cdc13-KK46AS-KK57AS-KKRR112ASSA-KKLKK155ASLAA-S177S-sfGFPcp:leu1+ plo1-mCherry:natR*	figure 7; electronic supplementary material, figure S7	NLS-KK mutant

Strains with *zfs1+::natR:P.adh31-tetR-tdTomato*, *dh1L::ura4+:tetO, cdc13-S177S-sfGFPcp, P.adh1-cdc13-S177S-sfGFPcp*, *cdc2-GFP*, *mad3-ymEGFP*, *cut2-GFP* and *plo1-mCherry* have been described previously [96–99]. Strains expressing—from an exogenous locus—only the N-terminus of Cdc13 (*cdc13(1-177)-sfGFPcp*) or Cdc13 NLS mutants were generated by cloning the respective fragments or mutant versions into a pDUAL vector [100] and integrating the vector at the *leu1* locus. The proper sequence of the vectors was confirmed by Sanger or Nanopore whole-plasmid sequencing. Some strains have a Y66L mutation introduced into GFP, which makes GFP non-fluorescent. This allows us to visualize other proteins with GFP while maintaining the tag for comparability with other strains. In circularly permuted sfGFPcp, Y138 (LTYGV) is the residue corresponding to Y66 (FTYGV) in canonical GFP.

### 4.2. Growth conditions

Cells were grown in Edinburgh minimal medium (EMM, MP Biomedicals, 411003) at 30°C to a concentration of 8 × 10^6^ – 1.5 × 10^7^ cells ml^−1^. Leucine (0.2 mg ml^−1^) or adenine (0.15 mg ml^−1^) was added when required. Preconditioned medium (made by filtering EMM cultures) was added when cultures were diluted to low densities.

### 4.3. Time-lapse imaging

Time-lapse imaging was conducted on a DeltaVision Elite microscope equipped with an Olympus 60×/1.42 Plan-APO oil objective, LED illumination and a PCO edge sCMOS camera. Cells were kept at 30°C for the duration of imaging (EMBL environmental chamber). Cells were either mounted in µ-Slide 8 well glass bottom chambers (Ibidi, 80827) coated with lectin (50 µg ml^−1^; Sigma-Aldrich, L1395) or loaded into microfluidics chambers (Millipore-Sigma, Y04C or Y04T) that had been washed with EMM and prewarmed to 30°C. Media flow for the microfluidics chambers was controlled using a CellAsic ONIX2 microfluidics system. EMM supplemented with 50% preconditioned medium was perfused at 2 psi. After being loaded into the viewing chamber of the microfluidics plate, cells were left to acclimate for 3 h before starting imaging. When imaging in µ-Slide chambers, cells were kept on the 30°C microscope stage for 15 min before starting imaging. Brightfield images were taken at the bottom or central slice of each field of view and fluorescence images were acquired using ‘optical axis integration’ (sum projection) over a 3.6 μm Z-distance. Fluorescence images in experiments shown in figures 2, 4, 6 and 7 were additionally deconvolved using SoftWoRx (GE Healthcare) software with three cycles of the ratio method (conservative).

### 4.4. Imaging of asynchronous cultures

Asynchronous cultures were either imaged at 30°C on a DeltaVision Elite microscope (see above) or at room temperature (approx. 22°C) on a Zeiss AxioImager M1 equipped with Xcite Fire LED illumination (Excelitas), a Zeiss Plan-APO 63×/1.4 oil objective and an ORCA-Flash4.0LT sCMOS camera (Hamamatsu). Cells were pelleted at 3300 rcf for 1 min, mounted from the pellet onto slides, covered with #1.5 glass coverslips and immediately imaged. Z-stacks were collected and the slice corresponding to the midplane of the cell was used for the quantification of nucleocytoplasmic ratios.

### 4.5. Image analysis

For the experiments in figures 1, 2, 5 and 6, and electronic supplementary material, figures S2 and S6, brightfield images were used to segment individual cells using the YeaZ neural network with custom weights [101]. Masks were manually edited using the YeaZ graphical user interface as needed. Cells partially out of frame were eliminated from the analysis. A custom Fiji [102] script was used to load corrected YeaZ cell masks as regions of interest (ROIs). Nuclei were segmented in Fiji using tdTomato-NLS fluorescence and Otsu thresholding. A Gaussian blur was applied to smooth the nuclear edges. After manual checking and correction, nuclear ROIs were assigned to cellular ROIs based on position; and size and fluorescence intensity were quantified. Cytoplasmic area and integrated intensity were calculated by subtracting nuclear area and integrated intensity from the respective whole cell measurements. To determine nucleocytoplasmic ratios, the fluorescence intensity outside cells, measured as the mean from several manually drawn ROIs, was subtracted as background. (Note that this does not take autofluorescence into account, which is difficult to quantify. Both nuclear and cytoplasmic concentrations are, therefore, slightly overestimated.) To calculate nucleocytoplasmic ratios, nuclear GFP concentration (integrated signal by area) was divided by the respective cytoplasmic concentration and normalized within each experiment to the mean nucleocytoplasmic ratio of cells expressing wild-type Cdc13-sfGFP.

The experiment in figure 4 lacked a brightfield image for cell segmentation and a fluorescent marker for nuclear segmentation. Cells were, therefore, segmented using trainable Weka segmentation [103] based on the diffuse Plo1-mCherry signal. Cytoplasmic concentrations were measured by manually placing two ROIs into the cytoplasm, one on each side of the nucleus. Plo1 enrichment at the SPB region and Cdc13 nuclear intensity were estimated by calculating the mean of the 40 brightest pixels in the cell. For the experiment in figure 7 and electronic supplementary material, figure S7, cells were segmented manually. Plo1 enrichment at the SPB region and nuclear Cdc13-sfGFP were estimated by calculating the mean of the 40 brightest pixels in the cell. Raw data curves were smoothed using the loess function in R (span 0.3).

The Pomegranate image analysis pipeline [104] was used for the three-dimensional quantification of Cdc13-sfGFP shown in electronic supplementary material, figure S1.

## Data Availability

Supplementary material is available online [105].
